# LCA of a Membrane Bioreactor Compared to Activated Sludge System for Municipal Wastewater Treatment

**DOI:** 10.3390/membranes10120421

**Published:** 2020-12-14

**Authors:** Dimitra C. Banti, Michail Tsangas, Petros Samaras, Antonis Zorpas

**Affiliations:** 1 Laboratory of Technologies of Environmental Protection and Utilization of Food By-Products, Department of Food Science and Technology, International Hellenic University, GR-57400 Thessaloniki, Greece; bantidim@gmail.com (D.C.B.); samaras@ihu.gr (P.S.); 2 Laboratory of Chemical Engineering and Engineering Sustainability, Faculty of Pure and Applied Sciences, Environmental Conservation and Management, Open University of Cyprus, Latsia P.O. Box 12794, Nicosia 2252, Cyprus; antonis.zorpas@ouc.ac.cy

**Keywords:** life cycle impact assessment, membrane bioreactor, conventional activated sludge, municipal wastewater treatment, environmental impact

## Abstract

Membrane bioreactor (MBR) systems are connected to several advantages compared to the conventional activated sludge (CAS) units. This work aims to the examination of the life cycle environmental impact of an MBR against a CAS unit when treating municipal wastewater with similar influent loading (BOD = 400 mg/L) and giving similar high-quality effluent (BOD < 5 mg/L). The MBR unit contained a denitrification, an aeration and a membrane tank, whereas the CAS unit included an equalization, a denitrification, a nitrification, a sedimentation, a mixing, a flocculation tank and a drum filter. Several impact categories factors were calculated by implementing the Life Cycle Assessment (LCA) methodology, including acidification potential, eutrophication potential, global warming potential (GWP), ozone depletion potential and photochemical ozone creation potential of the plants throughout their life cycle. Real data from two wastewater treatment plants were used. The research focused on two parameters which constitute the main differences between the two treatment plants: The excess sludge removal life cycle contribution—where GWP_MBR_ = 0.50 kg CO_2_-eq*FU^−1^ and GWP_CAS_ = 2.67 kg CO_2_-eq*FU^−1^ without sludge removal—and the wastewater treatment plant life cycle contribution—where GWP_MBR_ = 0.002 kg CO_2_-eq*FU^−1^ and GWP_CAS_ = 0.14 kg CO_2_-eq*FU^−1^ without land area contribution. Finally, in all the examined cases the environmental superiority of the MBR process was found.

## 1. Introduction

Wastewater treatment plants protect the environment [1,2] by solving the major issue of pollution caused by municipal and industrial wastewater. Since wastewater treatment processes constitute a significant environmental issue and affect the quality of freshwater and human health, their role is getting more and more important in the framework of Sustainable Development Goals [3]. Nevertheless, their operation has often been connected to several environmental impacts throughout their life cycle, such as emissions, soil and water pollution, and odor problems [4]. Energy consumption, use of chemical substances, land use and sludge production are some of the aspects of the processes of wastewater treatment plants that may harm the environment.

Membrane bioreactors (MBRs) embrace an advanced wastewater treatment process that has been developed during last 20–30 years [5,6]. According to recent research [7,8], it has been found that more than 2500 MBR plants worldwide have been constructed since 2008, and they present an annual growth rate equal to 10.5%. MBRs differ from the conventional activated sludge process as they replace the secondary settling tank by a membrane filtration unit [9,10]. MBRs present several distinct advantages compared to the conventional activated sludge process, like higher volumetric loading, excellent effluent quality, good disinfection capability depending on the membranes pore size, reduced footprint and sludge production, process flexibility towards influent changes and improved nitrification [11]. Furthermore, wastewater treatment plants using this novel technology occupy much smaller plot areas; they usually do not require additional chemicals in order to improve their efficiency and operate under less sludge retention time [12,13].

Several impact analysis and environmental performance improvement methods have been implemented to evaluate wastewater treatment processes, methods, systems, or plants until today. For instance, a multi-step simulation-based methodology and a scenario-based optimization approach were used to improve, through operational changes, the effluent quality and to reduce energy consumption of the biggest wastewater treatment plant in Italy [14]. Furthermore, in another research work a total environmental impact score was assessed to define the environmental impact of wastewater discharge, by considering the volume of wastewater and the quality of main processes [15].

The evaluation of the environmental impacts throughout the life cycle of wastewater treatment plants including MBR technology has been studied in several research papers [4,9,16,17,18,19,20]. LCA seems to be an appropriate environmental assessment tool [21,22] that can also streamline the decision-making process in the wastewater treatment industry [4] and be used to design and construct the most appropriate wastewater treatment plants [20]. LCA has been implemented either to assess and minimize the effects from wastewater treatment units [4,16] or even to evaluate how different weather conditions, such as comparison of wet and dry weather, affect the treatment parameters, in order to decide about the best configuration [23]. Moreover, comparison of alternative MBR configurations and selection of the most effective [9] has been carried out by the quantitative definition of the resources consumed and the estimation of the emissions produced during the construction, operation and end-of-life demolition of an MBR pilot unit [11].

Moreover, LCA has been used to compare three different treatment processes in combination with economic efficiency analysis, aiming to propose sludge-management alternatives in a large city [19]. The methodology has also been found appropriate for assessing the sustainability of wastewater treatment plant design in decentralized systems located in rural areas [24]. The environmental issues concerning the construction phase, by involving materials and transportation for civil works undertaken, has also been analyzed by using LCA and the critical role of building materials to the size of the impacts was highlighted [25]. Advanced wastewater treatment techniques for removal of pharmaceuticals and personal care products have also been assessed and relevant recommendations have been formed [26].

In several case studies of LCA of wastewater treatment plants, all stages of their life cycle have been studied, including their construction, operation, and demolition. On the other hand, several researches have been performed focusing only on the LCA of the process of wastewater treatment plants. Smith et al. (2014) [27] evaluated an anaerobic membrane bioreactor (AnMBR) technology in comparison with conventional wastewater energy recovery technologies, focusing only on the process. Furthermore, Wang et al. (2012) [28] focused on the process, aiming to create an evaluation scheme for choices of wastewater treatment processes that quantifies adverse environmental effects as well as bioenergy and nutrient recovery indices. Other researchers investigated different LCIA methods for a full-scale wastewater treatment plant focusing once again on the process [29].

There are several impact categories that have been used for LCA throughout the literature. Although, the energy consumption has concerned most of the studies, some of them, also, has evaluated greenhouse gas emissions, toxicity, and eutrophication [20]. Among the most-commonly calculated impact categories for wastewater treatment LCA has, the global warming potential, the eutrophication potential, the ozone depletion potential, the photochemical ozone creation potential, and the acidification potential are included [4,9,11,18,24,30,31]. Finally, these categories cover almost the whole range of environmental hazards that may be affected by the operation of wastewater processes, systems, and plants.

Summarizing, MBRs constitute a highly novel technology that has been widely used for wastewater treatment during last decades due to their various advantages. However, MBRs present a basic drawback, the membrane fouling problem that results in increased energy consumption due to the intense aeration of the membrane and consequently increased operating costs. On the other hand, conventional activated sludge process is an old, thoroughly tested, and therefore reliable and widely-used process. LCA method has been extensively used to improve wastewater treatment performance of either MBRs or conventional activated sludge processing. However, up to date limited studies have been conducted, focusing on the environmental impact comparison of the two wastewater treatment methods, aiming to select the optimal one in terms of their environmental footprint. This research work aims to address this issue, comparing the environmental impact of a membrane bioreactor unit and a conventional activated sludge unit that treat municipal wastewater of similar loading, giving simultaneously effluent of similar high quality. The research is further focused on two parameters which constitute the main differences between the two treatment plants: The production of excess sludge and the land area they occupy. The functional unit used in both cases is 1 m^3^ of effluent (treated wastewater).

## 2. Materials and Methods

### 2.1. The LCA Methodology

LCA is an environmental analysis method that examines the total life cycle of a process, system or product spotting their potential impacts to the environment [21,22]. It is standardized and addresses the environmental aspects from the acquisition of the raw material, through production, use, end-of-life treatment, recycling, and final disposal of a product [32]. According to ISO 14040:2006 [32,33], there are four phases that should be followed for a study: (1) The goal and scope definition phase, where the functional unit (FU), the system boundaries and the level of detail of the analysis are specified; (2) the life cycle inventory (LCI) analysis phase, where the collection of the necessary data for the studied system is performed; (3) the life cycle impact assessment phase (LCIA) that aims to gather information for LCI results by assessing the impacts, to identify their environmental importance, and finally, (4) the life cycle interpretation phase, during which the inventory and impact assessment phase results are recapped and discussed and conclusions and recommendations are formed. The LCA tools are widely used in wastewater treatment environmental analysis aiming to improve the environmental performance of goods and services, including products belonging to the agri-food sector [21], quarries [34,35], and finally they are considered as highly important tools for environmental impact assessment [36].

The present study follows the requirements of ISO 14040:2006 [32] methodology. Specifically, the goal and scope definition phase is consisted of Section 2.2 and Section 2.3, the LCI analysis phase is consisted of Section 3.1, Section 3.2, and Section 3.3, the LCIA phase is presented in Section 3.4 and Section 3.5 and the interpretation phase is included in discussion (Section 4) as well as in conclusions (Section 5) of the paper.

### 2.2. Goals and Scope

**Figure 1:**
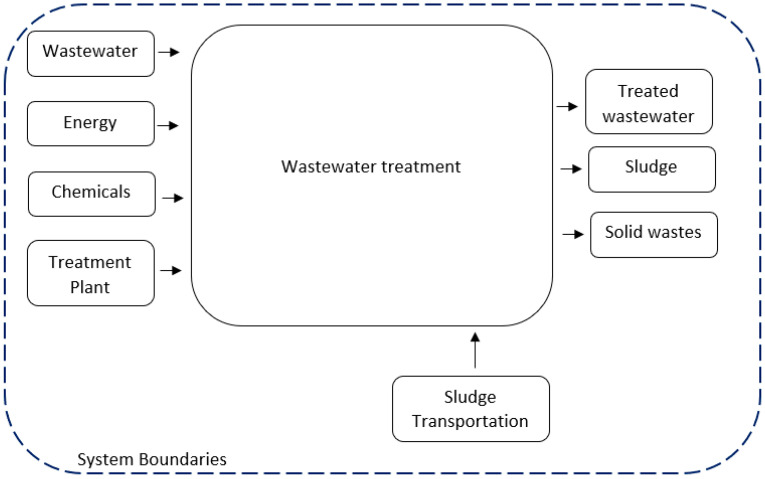
System boundaries.

The studied system covered all the wastewater treatment plant operation phases, i.e., influent wastewater, treatment processes and effluent production, including by-products, such as the sludge and membrane modules disposal. Energy consumption was also taken into consideration. An outline of the system boundaries is presented in Figure 1.

The gas pollutants that can be released directly from the wastewater treatment processes were not considered in the LCIA study. Gas pollutants are produced during the biological wastewater degradation process and are calculated based on the load of influent and effluent wastewater (defined by COD, TN, and SS concentrations) as well as the inflow and outflow rates, which are similar for both units [37,38]. Therefore, the gas pollutants were considered to be similar for the two compared units and therefore not taken into account.

The main solid wastes of the two units, which constitute one of their basic differences, was the excess sludge that was equal to 14 m^3^/day for the membrane bioreactor unit and 29 m^3^/day for the conventional activated sludge unit. Therefore, the excess sludge was taken into account at the LCIA study considering that it was transported with truck to a landfill over a distance of about 35 km for both cases. Moreover, the membrane modules of the MBR unit were considered as solid wastes as their lifespan was 5 years. They also transported to the landfill with truck, taking into account that they were constructed from polyethylene and their weight was 150 kg per membrane module, i.e., 450 kg for the 3 membranes used [39]. Regarding the equipment of the two units, such as stirrers and blowers, special attention was given to their proper maintenance and repair in case of damage and thus their average life span was contemplated to be greater than 20 years. Thus, they were not counted as solid wastes in the LCA study as they had a negligible effect on the final result. Other wastes, such as chemical packaging, were also considered that affect negligibly the overall environmental impact caused by each unit and therefore were not taken into account in the LCIA study.

#### 2.2.1. Functional Unit

The functional units (FUs) reflect a marketable product. In order to ensure that the input and output data are normalized in a mathematically consistent way, the functional units or/and reference flows have to be measurable and clearly determined [40]. Several FUs may be used for a wastewater treatment LCA, e.g., influent generated by one person equivalent [18], one population equivalent [24] or a per day inflow quantity [16]. For similar LCA studies [11,19], 1 m^3^ of influent wastewater gave a satisfactory analysis base. Nevertheless, for this study, 1 m^3^ of effluent (treated wastewater) is suitable and practical to be selected as the FU. This measure facilitates the data collection as well as the inventory formation and was used for both plants’ analysis.

#### 2.2.2. Impact Categories

Similar to other research practices [4,9,19,20,24] and aiming to cover all the potential impacts of the operation of the wastewater treatment units to the environment, including effects to soil, air, and water, five environmental impact categories were chosen to be calculated in the LCIA, for the two alternative units that were examined. These are the acidification potential (AP) measured in kgSO_2_-eq_FU^−1^, the eutrophication potential (EP) in kg PO_4_-eq_FU^−1^, the global warming potential (100 years) (GWP) in kg CO_2_-eq_FU^−1^, the ozone depletion potential (ODP) in kg CFC-11-eq_FU^−1^ and the photochemical ozone creation potential (POCP) in kg C_2_H_4_-eq_FU^−1^. All of them were specified according to the CML 2001 (April 2013 and January 2015 version) impact assessment method of the Centre of Environmental Science of Leiden University [41].

#### 2.2.3. Description of the Two Studied Wastewater Treatment Units

The LCA study was comparative between two separate wastewater treatment plants, the first of which used the membrane bioreactor (MBR) technology and the second one the conventional activated sludge process. Therefore, information and quantities for both units’ processes were collected according to their regular operation, in order to prepare the Life Cycle Inventory. The two units are shortly presented below.

The studied membrane bioreactor unit was located in North Greece and received municipal wastewater with Q_in_ = 528 m^3^/day, influent BOD (Biochemical Oxygen Demand) = 400 mg/L, influent TN (Total Nitrogen) = 60 mg/L and influent SS (Suspended Solids) = 440 mg/L. Wastewater entered the denitrification tank (V = 68 m^3^), where it was mixed with a stirrer, and an aeration tank followed, of active volume 145 m^3^, where the wastewater was aerated by diffusers. After that, the wastewater passed into a membrane tank of active volume 1050 m^3^ that contained 3 hydrophilic flat sheet ultrafiltration membrane modules (Microdyn) with nominal pore size 0.04 μm. The mixed liquor of the membrane tank was, also, aerated by diffusers. BOD and TN resulting from the permeate/effluent of the membrane bioreactor was less than 5 mg/L, and SS was found less than 1 mg/L. Wastewater outflow was equal to Q_out_ = 528 m^3^/day. At the same time, part of the mixed liquor of the membrane tank was recirculated to the denitrification tank while excess sludge was removed with Q_sludge_ = 130 kg/day that was transferred to a landfill.

The studied conventional unit with tertiary treatment was located in North Greece as well, and received municipal wastewater with Q_in_ = 528 m^3^/day, influent BOD = 400 mg/L, influent TN (Total Nitrogen) = 60 mg/L and influent SS (Suspended Solids) = 440 mg/L, similar to the membrane bioreactor unit. Firstly, the wastewater entered the equalization tank (V = 37 m^3^), where it was mixed with a stirrer. The unit included a denitrification tank followed by a nitrification tank with total working volume 650 m^3^, where the wastewater was mixed with a stirrer in the first tank and was aerated by diffusers in the latter. After that, the wastewater passed into a secondary sedimentation tank of 78.5 m^3^ active volume while tertiary treatment followed that was carried out in a system included a mixing tank—where PAC and polyelectrolyte were added as flocculants—a flocculation tank and a drum filter. In the mixing and flocculation tanks mixed liquor was stirred with stirrers. The effluent of the conventional unit had the same high quality as the MBR unit. Specifically, BOD and TN of the effluent was less than 5 mg/L and SS was found less than 1 mg/L. The outflow rate was Q_out_ = 499 m^3^/day. At the same time, part of the mixed liquor of the drum filter tank was recirculated to the denitrification tank. Moreover, excess sludge was removed from the secondary sedimentation tank with Q_sludge_ = 269.3 kg/day and was transferred to a sanitary landfill.

### 2.3. Software

In order to carry out the LCA studies and the corresponding calculations, the open source and free software openLCA (GreenDelta GmbH, Berlin, Germany), which was developed by GreenDelta [42] was used. This software offers the possibility to import many free as well as commercial LCA databases and Life Cycle Inventory Analysis (LCIA) methods, giving the ability to design a life cycle system by connecting all LCI elements and to quantify the LCIA according to the method chosen.

## 3. Results

### 3.1. Systems Modelling

**Figure 2:**
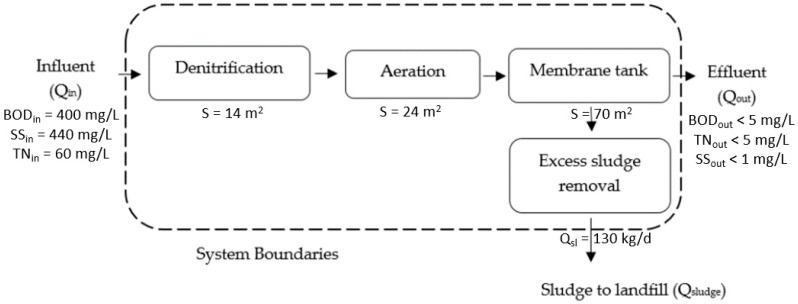
Membrane bioreactor unit system.

**Figure 3:**
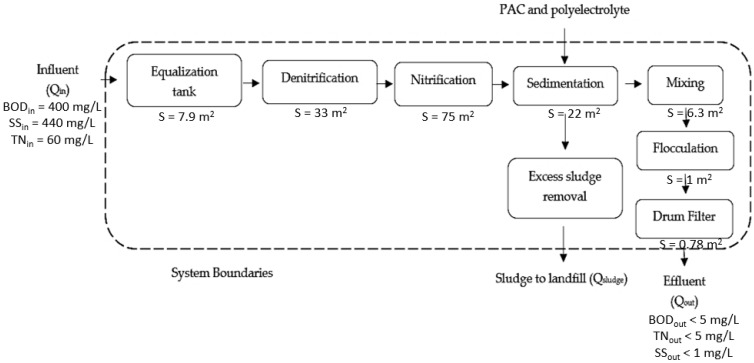
Conventional activated sludge unit system.

The system models, according to the LCA goal and scope of the units under comparison are presented for the membrane bioreactor unit in Figure 2 and for the conventional activated sludge unit in Figure 3. The two wastewater treatment units include a series of processes aiming to treat wastewater of the same load giving an effluent, also, of similar clarity. As it is presented in Figure 2 and Figure 3, the CAS unit follows a sequence of several processes in order to achieve the same high effluent quality with the MBR unit. It is, also, observed that the sub-units of the CAS occupy much larger land area compared to the MBR, while producing a large amount of excess sludge.

### 3.2. Life Cycle Inventory (LCI)

LCI recorded input/output data regarding the system being studied [32]. For both examined units the inventory included all the input and output data recorded according to the daily operation of the units. Furthermore, all the materials used and required energy data per day among the lifetime of the units were included. Secondary data concerning the background system, e.g., electricity, chemical substances and transportation, were received from the LCI databases, EcoSpold 2 and product environmental footprints (PEF). In detail, electricity life cycle data were taken from PEF database for Electricity EU-28+3. Maintenance of the units was not included in the inventory, since there were not sufficient data available. Finally, Table 1 presents the analytical LCI of the membrane bioreactor unit, whereas Table 2 presents the LCI of the conventional activated sludge unit.

### 3.3. Limitation of the Study and Data Quality

In-situ surveys were used for the collection of all the primary data used for the LCA for both units, while secondary data were drawn from well-established LCI databases. The quality assessment of the data for the LCI is presented in Table 3 according to the ecoinvent guidelines [43]. Five indicators were used: Reliability, completeness, temporal correlation, geographical correlation, and further technological correlation, characterized in five quality levels from one to five. The score of the highest quality level is 1, where the data are specified for the particular case study, and for the lowest level is five, where the data comes from unknown source or non-qualified estimation [43].

Although the primary data acquired from the in-situ survey are of high quality, the secondary data, mainly concerning the background systems, e.g., production of chemicals or membranes, could be improved. In this case similar data were used, as no information, regarding the exact elements used, were detected in the available databases or in local LCI.

### 3.4. Life Cycle Impact Analyses (LCIA)

The Life Cycle Impact categories for the two wastewater treatment plants have been calculated using the openLCA software. The CML (baseline) [v4.4, January 2015] impact assessment method was used and the size of the impacts of each category as calculated for both units are presented in Table 4.

**Figure 4:**
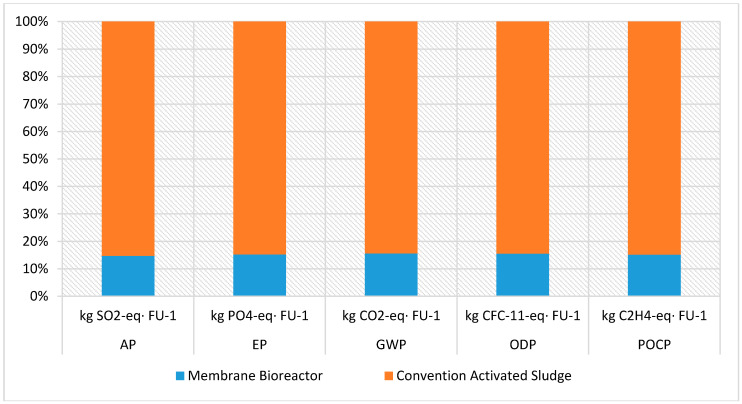
Comparative LCIA for the two units.

The calculated Life Cycle Impacts (LCIA) results are, also, presented in Figure 4, comparing the two wastewater treatment units.

### 3.5. Contribution Differences

**Figure 5:**
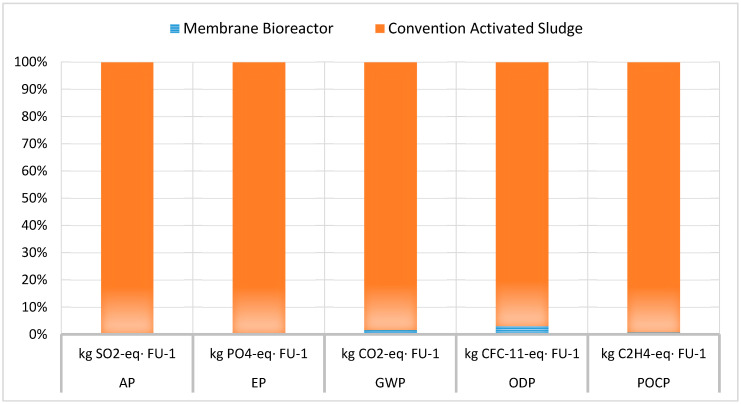
Comparison of the LCIA for the two units with no plant contribution.

Several elements, where the two wastewater treatment technologies under study differed significantly, affected the impact categories size. Those included the use of chemicals, the use of membranes, the size of the units and the daily produced sludge. As the use of membranes and chemicals were not common elements for the two alternative processes, they were not further compared in this study. Nevertheless, as the units’ size and sludge removal were common elements for the two plants, their contribution to the LCIA was further compared. Therefore, Table 5 and Figure 5 presents the LCIA of both units for the case where the contribution of the wastewater treatment plant, i.e., premises and land use, was not included.

**Figure 6:**
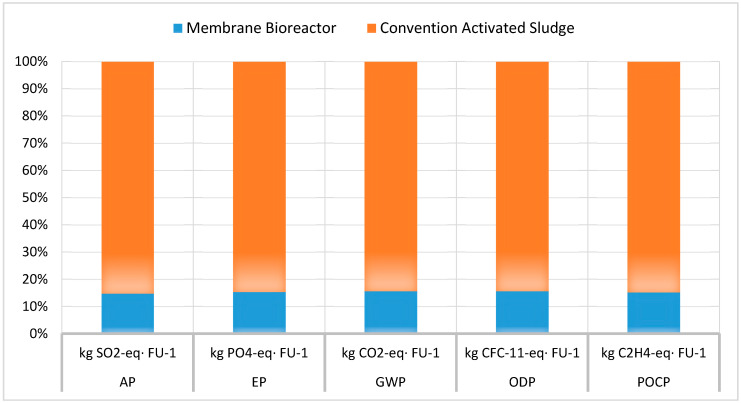
Comparison of LCIA for the two units with no sludge removal contribution.

Table 6 presents the LCIA of both units with wastewater treatment plant contribution but without the contribution of the sludge transportation and disposal to landfill. Subsequently, Figure 6 shows the comparison of the impact categories size for this case.

## 4. Discussion

Limited LCA studies are available with similar goals and scope including the specific FU of the present one, of 1 m^3^ effluent wastewater. Nevertheless, analysis regarding 1 m^3^ of influent wastewater [11,19] are available. According to [11], a different LCIA method was used and it was found, for example, that 4.65 kg CO_2_-eq/m^3^ were produced for every FU treated by a pilot MBR unit, a number that differs significantly from 0.496 kg CO_2_-eq/m^3^ per FU calculated by the present study. This could be explained either by the different method used or because the present study is for a unit in operation. On the other hand, the results of [19] cannot be comparable with the current study, since a different approach was implemented. Studies for other FUs are difficult to be compared or used for validation of the current results and therefore, the results of this study are solely interpreted.

Regarding the quality of the secondary data used for this study, the following are worth commenting on, as some elements of the LCI data came from two different databases. According to the ecoinvent guidelines and especially Indicator Score Table 10.4 [43], the reliability of the secondary data matched to the lowest quality, because they were considered as a non-qualified estimate. Moreover, since there were no available data for some elements, such as the polyethersulfone membrane module, some of them were replaced with similar elements, for example with polyethylene membrane module, as presented in the LCI tables. Moreover, data for Greece, where the units under study operate, were also not available, so data from Europe were used, and therefore the geographical correlation was assessed with the second lowest score, i.e., for slightly similar production conditions. Further technological correlation indicator was assessed with the same low score, as data on related process or material were used. Their completeness as well as their temporal correlation were assessed with the medium score. The first because only representative data were used and the latter because the time period of the data was unknown, but for sure it was less than 10 years. In any case, an uncertainty analysis would better present the data quality, but some prices are required, which due to their unavailability will be estimated approximately, without giving any substantial advantage to the final conclusion.

It is established that the membrane bioreactor wastewater treatment technology has various advantages over the conventional activated sludge process [9,11]. However, MBRs consume more energy and therefore they have increased operating costs due to the membrane fouling problem [12,13]. However, there is insufficient data regarding their environmental impact, an issue that is investigated in this research work. Therefore, according to the abovementioned LCI and LCIA results (Table 4, Figure 4), MBR’s environmental impact was significantly lower than that of the conventional method. A result attributed to the fact that, according to the inventory analysis, besides no chemicals were used in the MBR case, significantly smaller plot area required, including premises and land area, as well as less sludge—entailing handling and disposal—was produced, as it is, also, presented in Figure 2 and Figure 3. These advantages of the MBR unit fully justify its better environmental impact compared to the conventional wastewater treatment unit.

It is worth noting that this work did not study the comparison of energy consumption between the two units but only their environmental impact during their operation. The higher environmental impact of the CAS unit compared to the MBR is largely attributed to the fact that in this study there is not a comparison of an MBR unit with a simple CAS unit consisting of an aeration tank and a sedimentation tank. Instead, the CAS unit includes a sequence of processes, such as equalization, denitrification, nitrification, sedimentation, chemical mixing, flocculation, and drum filter aiming to achieve similar high-quality effluent with the MBR unit. Each of these sub-processes burdens further the environment during its operation, for example consuming further energy for the operation of the mixers and the blowers that operate 24 h per day or/and with chemicals addition. For all these reasons, it is concluded that CAS puts more strain on the environment during its operation comparing to the MBR, trying to achieve similar high effluent quality.

According to [41], the major acidifying pollutants for AP impact category are SO_2_, NO_x_, and NH_x_. For EP, the excessively high environmental levels of macronutrients like nitrogen (N) and phosphorus (P) are significant. For ODP, major issue represents the emissions of CFC and Halon. Moreover, among others, toluene, trans–2-Butene, trans–2-Hexene, and trans–2-Pentene are important for POCP. GWP is depended on GHG emissions to the air throughout the full process. Based on the LCIA results, it seems that these impact contributors were produced in greater quantities by the traditional activated sludge technology during the life cycle of the units under study.

The results presented in Table 5 and Table 6 and the relevant Figure 5 and Figure 6 are crucial for the comparison of the environmental impact of the two processes. Since a major advantage of MBR units was the smaller required plant, as it can be concluded by the large number of processes required by the CAS (Figure 2 and Figure 3), this could be deemed to be one of the main factor that affect the size of the environmental impacts. Nevertheless, when the wastewater treatment plants life cycle for both cases was excluded, the LCIA showed that the membrane bioreactor unit had better environmental performance once again, as it is presented in Table 5 and Figure 5. Moreover, taking into consideration that the membrane bioreactor technology produced less sludge for the same influent load, a question was raised, related to the potential of this advantage for affecting the impacts size. The results of the LCIA for the two units excluding the sludge removal process in both cases (Table 6 and Figure 6), showed that the conventional unit’s impact was still worse than the MBR’s. These observations could be interpreted that the use of MBR process itself is still more environmentally friendly compared to the conventional one, when they treat municipal wastewater of similar loading and they achieve similar high-quality effluent. Furthermore, another factor that may aggravate the environmental impact of the MBR unit, which is the life cycle of the membranes themselves, according to all the above mentioned LCIA results seems not to affect the MBR’s environmental superiority.

Consequently, the membrane bioreactor technology has to be further promoted, as besides their obvious advantages, according to this study their environmental impacts were, also, significantly smaller. The only aspect that was emerged by LCI for MBRs and seems that can be further improved is the handling of the membranes throughout their life cycle, including raw material use, manufacturing and disposal after the end of their life cycle.

## 5. Conclusions

Membrane bioreactors constitute a novel wastewater treatment technology, connected to several advantages compared to the conventional activated sludge process. However, they consume more energy and therefore they have increased operating costs due to the membrane fouling problem. This research work investigated the comparative environmental impact of the two processes for which incomplete data is available. A comparative LCIA study of two alternative plants, a membrane bioreactor unit and a conventional activated sludge unit, with same influent loading, in the form of ΒOD, TN, and SS, was performed and similar high-quality effluent, calculating five impact categories. AP, EP, GWP, ODP, and POCP were measured and it was found that they were significantly lower for the MBR unit. Moreover, excluding elements that was assumed to give significant advantage to the environmental impact of the MBR technology, such as land area and premises, and excess sludge production, from the LCIA, it was found once again that the MBR process itself was more environmental-friendly. Therefore, by this point of view their application is highly suggested instead of the use of the conventional activated sludge method.

## Figures and Tables

**Table 1:** Membrane bioreactor unit life cycle inventory (LCI).

Element	Input	Output	Quantity/Day	Quantity /FU	Unit	Notes
Sewage Treatment Unit	Capacity 1.9 × 10^8^ L/year (20 years lifetime)		5.21 × 10^5^	1.01 × 10^−7^	items	Ecospold 2 1/5 of conventional unit size
Wastewater Q_in_	528 m^3^/day		528	1.03	m^3^	
Influent Pump Electricity	7.67 kW × 7.34 h/day		56.3	1.10 × 10^−1^	kWh	
Denitrification Mixer Electricity	1.2 kW × 24 h/day		28.8	5.60 × 10^−2^	kWh	
Aeration Blower Electricity	10 kW × 24 h/day		240	4.67 × 10^−1^	kWh	
Membrane Blower Electricity	12 kW × 24 h/day		288	5.60 × 10^−1^	kWh	
Membranes [39]	450 kg		0.25	4.80 × 10^−4^	Kg	Polyethylene instead of Polyethersulfone (PES), 5 years lifetime
Sludge Circulation Pump Electricity	4 kW × 24 h/day		96	1.87 × 10^−1^	kWh	
Sludge Removal Pump Electricity	0.25 kW × 6 h/day		1.5	2.92 × 10^−3^	kWh	
Diffuse Pump Electricity	0.60 kW × 24 h/day		14.4	2.80 × 10^−2^	kWh	
Q Sludge Out	14 m^3^/day	130 Kg/day	130	2.53 × 10^−1^	Kg	To landfill
Sludge Transportation	35 Km × 14 m^3^/day		4550	8.85	Km Kg	Lorry EURO6 10 tons
Treated Wastewater Q_out_		514 m^3^/day	514	1.00	m^3^	

**Table 2:** Conventional activated sludge unit LCI.

Element	Input	Output	Quantity/Day	Quantity/FU	Unit	Notes
Sewage Treatment Unit	Capacity 1.9 × 10^8^ L/year (20 years lifetime)		2.60 × 10^−4^	5.22 × 10^−7^	items	EcoSpold 2
Wastewater Q_in_	528 m^3^/day		528	1.06	m^3^	
Influent Pump Electricity	7.67 kW × 7.34 h/day		56.3	1.13 × 10^−1^	kWh	
Equalization Tank Mixer Electricity	1.2 kW × 24 h/day		28.8	5.77 × 10^−2^	kWh	
Denitrification Mixer Electricity	1.2 kW × 24 h/day		28.8	5.77 × 10^−2^	kWh	
Nitrification Blowers Electricity	2 × 10 kW × 24 h/day		480	9.62 × 10^−1^	kWh	
Sedimentation Bridge Electricity	0.75 kW × 24 h/day		18	3.61 × 10^−2^	kWh	
Sludge Circulation Pump Electricity	4 kW × 24 h/day		96	1.92 × 10^−1^	kWh	
Sludge Removal Pump Electricity	0.25 kW × 6 h/day		1.5	3.01 × 10^−3^	kWh	
Q Sludge Out		269.3 Kg/day	269.3	5.40 × 10^−1^	Kg	to landfill
Sludge Transportation	35 Km × 29 m^3^/day		9425.5	1.89 × 10^1^	Km Kg	Lorry EURO6 10 tons
PAC (flocculent)	90.6 kg/day		90.6	1.82 × 10^−1^	Kg	EcoSpold 2 iron(II) chloride
Polyelectrolyte	0.966 kg/day		0.97	1.94 × 10^−3^	Kg	EcoSpold 2 aluminium sulfate, powder
Mixer Electricity	0.41 kW × 24 h/day		9.84	1.97 × 10^−2^	kWh	
Polyelectrolyte Pump Electricity	0.40 kW × 24 h/day		9.6	1.92 × 10^−2^	kWh	
Flocculation Mixer Electricity	0.41 kW × 24 h/day		9.84	1.97 × 10^−2^	kWh	
Drum Filter Electricity	0.50 kW × 6 h/day		3.0	6.01 × 10^−3^	kWh	
Treated Wastewater Q_out_		499 m^3^/day	499	1.00	m^3^	

**Table 3:** LCI quality assessment.

Assessment Indicator	Indicator Score Table 10.4 [43]	
	Primary Data	Secondary Data
Reliability	2	5
Completeness	1	3
Temporal Correlation	1	3
Geographical Correlation	1	4
Further Technological Correlation	1	4

**Table 4:** Life Cycle Inventory Analysis (LCIA) results.

Wastewater Treatment Unit	AP kg SO_2_-eq∙FU^−1^	EP kg PO_4_-eq∙FU^−1^	GWP kg CO_2_-eq∙FU^−1^	ODP kg CFC-11-eq∙FU^−1^	POCP kg C_2_H_4_-eq∙FU^−1^
Membrane Bioreactor	1.98 × 10^−3^	8.60 × 10^−4^	4.96 × 10^−1^	3.13 × 10^−8^	1.50 × 10^−4^
Convention Activated Sludge	1.15 × 10^−2^	4.77 × 10^−3^	2.68	1.70 × 10^−7^	8.40 × 10^−4^

AP: Acidification Potential, EP: Eutrophication Potential, GWP: Global Warming Potential, ODP: Ozone Depletion Potential, POCP: Photochemical Ozone Creation Potential.

**Table 5:** LCIA with no wastewater treatment plant life cycle contribution.

Wastewater Treatment Unit	AP kg SO_2_-eq∙FU^−1^	EP kg PO_4_-eq∙FU^−1^	GWP kg CO_2_-eq∙FU^−1^	ODP kg CFC-11-eq∙FU^−1^	POCP kg C_2_H_4_-eq∙FU^−1^
**Membrane Bioreactor**	6.23 × 10^−6^	1.94 × 10^−6^	2.35 × 10^−3^	3.65 × 10^−10^	3.83 × 10^−7^
**Convention Activated Sludge**	1.32 × 10^−3^	3.40 × 10^−4^	1.38 × 10^−1^	1.12 × 10^−8^	5.62 × 10^−5^

**Table 6:** LCIA with no sludge removal life cycle contribution.

Wastewater Treatment Unit	AP kg SO_2_-eq∙FU^−1^	EP kg PO_4_-eq∙FU^−1^	GWP kg CO_2_-eq∙FU^−1^	ODP kg CFC-11-eq∙FU^−1^	POCP kg C_2_H_4_-eq∙FU^−1^
**Membrane Bioreactor**	1.98 × 10^−3^	8.60 × 10^−4^	4.96 × 10^−1^	3.13 × 10^−8^	1.50 × 10^−4^
**Convention Activated Sludge**	1.15 × 10^−2^	4.77 × 10^−3^	2.67	1.70 × 10^−7^	8.40 × 10^−4^
